# L1000 Viewer: A Search Engine and Web Interface for the LINCS Data Repository

**DOI:** 10.3389/fgene.2019.00557

**Published:** 2019-06-14

**Authors:** Aliyu Musa, Shailesh Tripathi, Matthias Dehmer, Frank Emmert-Streib

**Affiliations:** ^1^Predictive Society and Data Analytics Lab, Faculty of Information Technology and Communication Sciences, Tampere University, Tampere, Finland; ^2^Institute of Biosciences and Medical Technology, Tampere, Finland; ^3^Institute for Intelligent Production, Faculty for Management, University of Applied Sciences Upper Austria, Linz, Austria; ^4^Department of Mechatronics and Biomedical Computer Science, UMIT, Hall in Tyrol, Austria; ^5^College of Computer and Control Engineering, Nankai University, Tianjin, China

**Keywords:** gene expression, big data, pharmacogenomics, web application, visualization, data science

## Abstract

The LINCS L1000 data repository contains almost two million gene expression profiles for thousands of small molecules and drugs. However, due to the complexity and the size of the data repository and a lack of an interoperable interface, the creation of pharmacologically meaningful workflows utilizing these data is severely hampered. In order to overcome this limitation, we developed the L1000 Viewer, a search engine and graphical web interface for the LINCS data repository. The web interface serves as an interactive platform allowing the user to select different forms of perturbation profiles, e.g., for specific cell lines, drugs, dosages, time points and combinations thereof. At its core, our method has a database we created from inferring and utilizing the intricate dependency graph structure among the data files. The L1000 Viewer is accessible via http://L1000viewer.bio-complexity.com/.

## 1. Introduction

We are living in the era of big data that sparked the establishment of the field data science (Smith, 2006; Ma'ayan et al., 2014; Jin et al., 2015; Emmert-Streib and Dehmer, 2019). For genomics, the recent growth of high-throughput biomedical and pharmacogenomic data (Edgar et al., 2002; Barrett et al., 2013; Woo et al., 2015; Musa et al., 2017) presents opportunities and at the same time challenges for their analysis. Paramount to these problems is ensuring that comparative genomics tools keep pace with the rate at which the data are produced (Tripathi et al., 2014; Smirnov et al., 2016; Stupnikov et al., 2016). A major challenge researchers are facing practically when interacting with “big data” is that most of the relevant information requires a considerable amount of time to subset, preprocess and obtain. Therefore, novel approaches for finding, selecting and downloading specific subdata from large data repositories are required. This is particularly a problem for obtaining raw data (Musa et al., 2018).

One example for such a big data repository is the Library of Integrated Network-based Cellular Signatures (LINCS) (Subramanian et al., 2017). The LINCS L1000 data repository consists of almost two million individual files containing information about the gene expression and metadata of cell lines perturbed by chemicals of certain dosages and durations (Vempati et al., 2014). While there are several desktop or command line software tools available that are capable of processing and manually extracting subsets of large data, these tools require software installation, which can be difficult and time consuming, and are only capable of processing the data locally (Duan et al., 2016; Enache et al., 2017; Fallahi-Sichani et al., 2017). Therefore, the datasets in the repository can only be analyzed if the end-user has specialized software installed. Improvements in software development but also web-based application technologies such as the Node.js and Vue.js JavaScript libraries, have led to the development of advanced web-based applications with animated and interactive features. While there are several interactive web-based tools that can access data via an application programming interface (API) (Subramanian et al., 2017), most of these tools have limited interactivity and sharing capabilities, e.g., by embedding them within web applications such as CMAP (Lamb et al., 2006). Furthermore, they are lacking an integration with biology specific analysis methods, e.g., for performing an enrichment analysis (Rahmatallah et al., 2017). Importantly, all of these tools operate on the signature level of the LINCS data, not the raw data. That means, if a user wants to select a specific subset of raw data for a dedicated analysis, there is no help available.

In order to facilitate the access and subset of raw data from the LINCS data repository we developed the L1000 Viewer. Our software is an interactive web application that does not require the user to install dedicated software, but it operates via any web browser on any operating system. Hence, it is operating system independent. Our web application provides a web interface with access to a dedicated database we created. This database utilizes the graph dependency structure between the individual data files of LINCS because *individual* does not mean *independent*. Specifically, the dependency structure is induced by the experimental conditions of the expression profiles and can be represented as a graph or network (Musa et al., 2018). In this graph, nodes correspond to data files and two data files are connected if they share experimental conditions. Our web application provides an easy-to-use interactive platform allowing the user to select subsets of raw data files that belong to specific forms of perturbation profiles, e.g., for specific cell lines, drugs, dosages and time points. This retrieval of data files is efficient and fast because of the utilization of the precomputed graph structure of the data files. In addition, we are providing software for a graphical summarization of the selected data showing various distributions of experimental parameters, e.g., sample sizes per cell line, sample sizes per concentration and sample sizes per time point. This provides valuable information for the user regarding the experimental design (Hinkelmann and Kempthorne, 2008) of follow-up computational pharmacogenomics studies based on these data.

Our paper is organized as follows. In the next section, we discuss all methods and data we use for our analysis. In the results section, we present our findings and provide results for an example application of our software. In the following sections, we discuss our results in detail. The paper finish with conclusions and an outlook.

## 2. Methods

### 2.1. LINCS Data

The LINCS data is a vast collection of gene expression profiles that includes many experimental samples covering more than seventy human cell lines. These cell lines are populations of cells that descended from an original source cell and having the same genetic make-up. These cells have been kept alive by growing them in a culture separate from their original source (Ong et al., 2017).

Specifically, LINCS contains about 1, 328, 098 gene expression profiles as a result from applying 42, 553 perturbagens (19, 811 small molecule compounds, 18, 493 shRNAs, 3, 627 cDNAs, and 622 biologics) for a total of 476, 251 signatures (consolidating replicates) (Subramanian et al., 2017).

### 2.2. Metadata and Data Standards

LINCS provides an API to annotations and perturbational signatures in the L1000 data repository via a collection of HTTP-based RESTful web services. An example of such a services is the Cell Service which is a service that describes meta-information for cell lines. Table 1 lists all the API services provided by LINCS for querying the L1000 metadata. These services support complex queries via simple HTTP GET requests that can be executed in a web browser or within most programming languages.

**Table 1:** List of available LINCS L1000 metadata APIs.

**Service (API)**	**Description**	**URL link**
Cell	The cell information service provides cell line meta-information for used in the experiments.	https://clue.io/api#cells
Gene	The gene information service returns meta-information for measured and inferred genes in the LINCS dataset.	https://clue.io/api#genes
Profile	The profile information service returns meta-information for instances in the LINCS dataset.	https://clue.io/api#profiles
Pert	The pert information service returns meta-information for perturbagens in the LINCS dataset.	https://clue.io/api#perts
Plate	The plateInfo service returns plate information.	https://clue.io/api#plates

### 2.3. Development of the Web Application

L1000 Viewer, the web application we have developed, consists of three main parts namely; (I) the database, (II) back-end, and (III) front-end implementations.

First, in order to store the data in the back-end, we use a MongoDB database. We convert and store all the raw data into a json object structure to enable identifier reference to each profile sample in the database. This enables the data to be stored as a document-oriented structure that allows fast user queries. The document-oriented model maps to the data objects in the application code in the back-end, making the data easy to work with. The MongoDB is a distributed database at its core, therefore, it enables a horizontal scaling, high availability and faster access.

**Figure 1:**
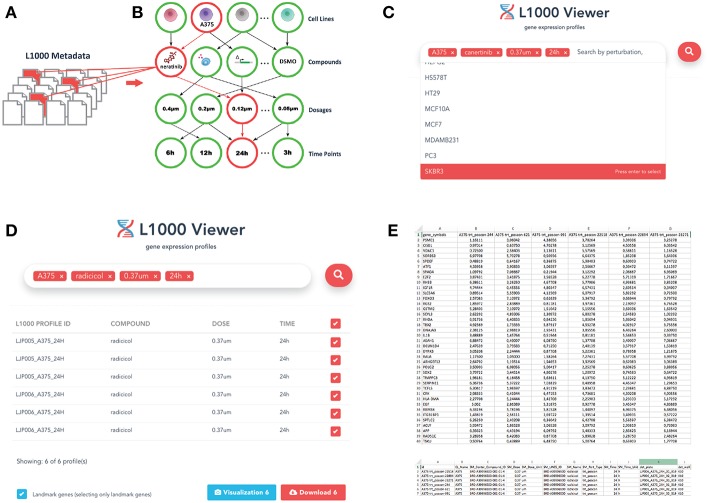
Overview of the L1000 Viewer. **(A)** Individual files forming the LINCS data. **(B)** Our graph database inferred from the relations among the individual profiles. **(C)** The interface allowing to enter queries to search the graph database. Shown is an example query discussed in the text. **(D)** The result of a query leads to a downloadable list. **(E)** Gene expression and metadata csv files.

The specific document structure is constructed from the experimental conditions of the individual data profiles within the LINCS data repository. As a result we obtained a relational representation of the documents using Mongoose schema. Mongoose provides a straight-forward, schema-based solution to model json object data into relationships. It includes built-in type casting, validation, query building, and logic hooks environment that wraps the Node.js native driver. This is visualized in Figures 1A,B. By (I) identifying and (II) utilizing this structure, our L1000 Viewer is able to efficiently provide a list of result profiles corresponding to an user-defined query. For instance, querying for the cell line A375, the drug neratinib, a dosage of 0.12μ and a duration of 24 h (see Figure 1C) results in 5565 files (see Figure 1D) that match the query list. That means the L1000 Viewer is a search interface that represent a relational structure from the underlying individual profiles corresponding to the instances in the database collection and allows by this an efficient querying of these profiles.

Second, for the back-end component, we decided to use Node.js for the server side architecture. A Node.js server environment was utilized to interact with the database through custom object-data modeling (ODM) calls adopted from pseudo relational database representation in Mongoose API. The main benefit of using this model is that you can define schemas for your collections which are then enforced at the ODM layer by architecture. It also has utilities for simplifying Node's callback patterns that make it easier to work with than the standard MongoDB driver alone. In general, this approach makes it even easier to use MongoDB with Node.js. Node.js is a web application development framework that uses convention over configuration. This means it can be efficiently used to spin a back-end development environment and also allows users to quickly understand the source code and contribute to development. It also supports a rich database of user-contributed libraries called packages that ease many complicated tasks, e.g., in handling downloading and archiving requests on the server side. We use packages such as backbone.js, archiver.js, underscore.js etc. to build the back-end. The L1000 Viewer was deployed on a Linux operating system supported by the Node.js runtime library. It is deployed on an Nginx server using Linode node.

Third, for the work-flow designer on the front-end we used javascript. Specifically, we use Vue.js to created the front-end representation. Vue.js is a widely used javascript framework and the L1000 Viewer uses it for handling all client side user interactions. The connections between the components of the interface are implemented using Vue.js plugins. It provides a mechanism to display and render the structural components from HTML tags. To interactively display the large collection of drug-induced profiles, the HTML5 elements were used to layout the profiles systematically.

Overall, the model-view-controller (MVC) software architecture was used to integrate the front-end, back-end and the database. The MVC pattern of design describes the behavior of the application's data, logic, rules, and generates an output based on changes to the application. The advantage of this is it helps in focusing on a specific part of the application name, the ways information is presented to and accepted from, the user.

### 2.4. Graphical Summary

In addition, we provide a functionality for an interactive visualization for viewing the selected profiles on the web. A user can click on the visualization button from the search results to visualize the selected profiles in different plots (e.g., boxplot representation of the profiles etc.). The metadata information of the selected profiles are also displayed. We provide R scripts for further metadata visualizations. Specifically, we provide scripts that allow the user to generate graphical summary statistics of their metadata query results. From the download function, the user can immediately download the profiles and use the R scripts on the subset of the data that was retrieved.

## 3. Results

We start this section by describing the basic functionality of the L1000 viewer web application we developed. Then we discuss its specific components in detail and provide an example.

### 3.1. General Overview of the L1000 Viewer

The L1000 Viewer has an interface allowing the user to enter queries in a disjunctive normal form (DNF), i.e., one can search for the simultaneous presence of search terms in the form,

(1) $$\begin{array}{r} {\text{term}}_{1}\text{ AND }{\text{term}}_{2}\text{ AND }\cdots \text{ AND }{\text{term}}_{n} \end{array}$$

For instance, in Figure 1C we entered the cell line A375, the drug neratinib, a dosage of 0.12μ and a duration of 24 h resulting in all profiles that are simultaneously indexed by cell line A375 AND drug neratinib AND a dosage of 0.12μ AND a duration of 24 h. The user can obtain a comprehensive list of available options directly from the L1000 interface by selecting the search field with a mouse click. This will open a pull-down menu that lists all available options that can be used as a search term in the query. Overall, the major categories for a query are cell lines, drugs and small compounds, dosages and time points.

A query finds any entity that exists among the treatment and control profiles. All queries will return a table of profiles listing unique ID numbers (e.g., LINCS profile ID, Compound), and if selected, a listing of metadata associated with the experiment will also be included in the download link. The interface is the data access point into the L1000 data repository.

The result from a query may be downloaded as a matrix of gene expression profiles. The array contains, for every gene, a binary vector representing the probe signal from the gene expression experiments (Subramanian et al., 2017). We converted the probe IDs to gene symbols for global representation. The L1000 Viewer allows the user to download complete matrices in either .csv or .csv.gz format, conferring flexibility to choose among alternative software analysis packages with optimal criteria and easy matrix subseting.

### 3.2. Constituting Components

#### 3.2.1. Data Available for Download

A large collection of almost two million L1000 gene expression profile data can be downloaded from the web interface, including the aforementioned GSE70138 from the LJP, CPC and CPD data repositories. Our application provides an easy-to-use and user-friendly interface to query the data repository, simply by searching for the desired experimental conditions.

From the L1000 Viewer web interface, metadata attributes can be used as input keyword to query the data repository. Any metadata associated with the input search can be entered in the search box. By default, the section provides four input fields for metadata: Cell, Perturbation, Dosage, and Time Point (Figure 1). Users can add new search terms for specific types of metadata by typing in the search box or remove one by clicking the close (x) sign on the right hand side of each keyword. The tag field is used to enter the keywords which are most descriptive of the input metadata.

### 3.3. Search Input

**Figure 2:**
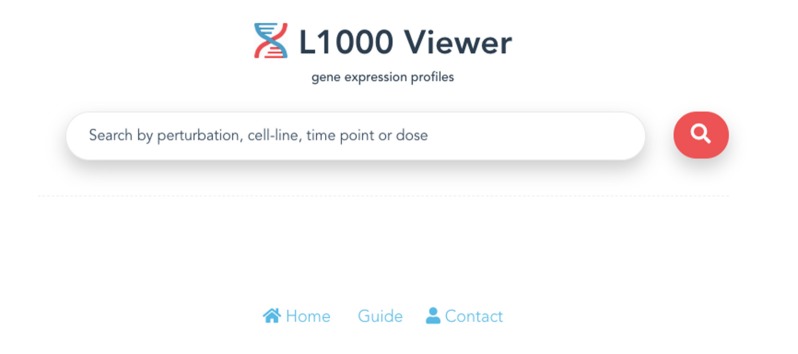
The entry point of the L1000 Viewer application.

**Figure 3:**
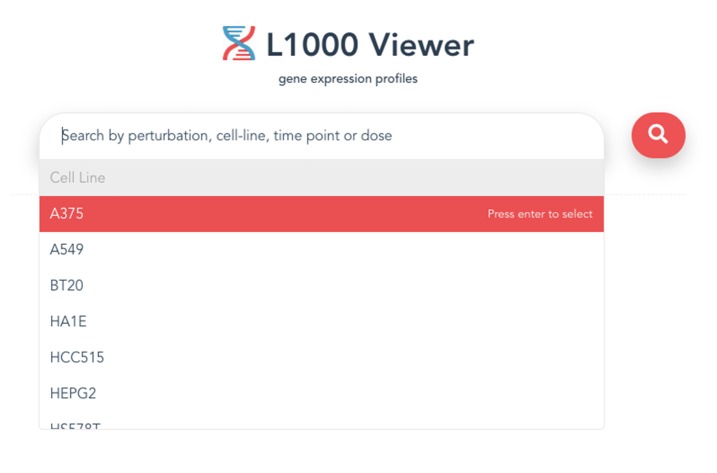
An example of a search term that can be used to search for gene expression profiles.

The entry point for our L1000 Viewer is to input a search term or a list of metadata query terms in the search box (see Figure 2) or paste a symbol (see Figure 3) into the search box. In order to provide guidance for setting search parameters, a query term is a list of cell lines, drug compounds, dosages or time points. The search button will only become enabled when the text box is filled with a search term, or when the text box is filled with a selection from the drop down list. By clicking the search button, the information for the top 50 samples will be displayed in a table below the search box. The interface provides the user with a user-friendly scrolling functionality for displaying more than 50 results.

### 3.4. Search Results

When a user successfully submits a query, the application will search and retrieve the corresponding profiles that match the user's input term and display the results. The performance of the search results will depend on the user-defined input terms. However, for any given query the application will guarantee fast results within milliseconds. In contrast, when the data is manually processed and retrieved directly from the LINCS data repositories a similar process can consume up to one day.

### 3.5. Download View

**Figure 4:**
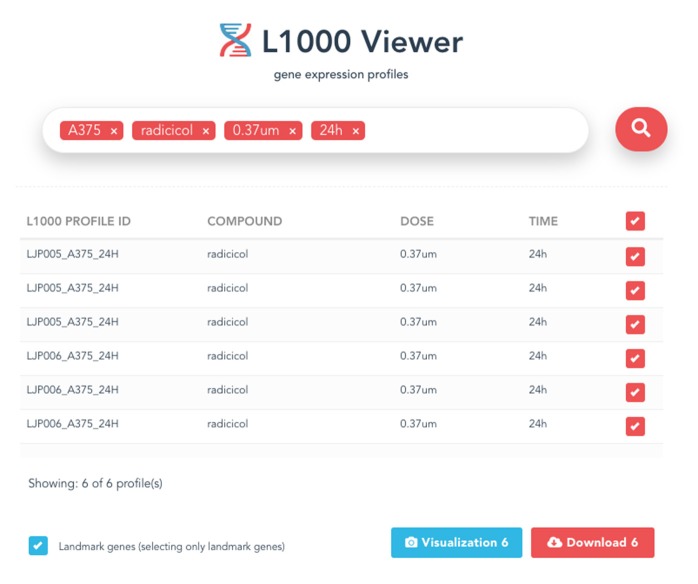
An example of the displayed search results using all four metadata information.

After the search results are displayed, the user can select individual profiles in the search results using the check box to fine-tune the results or decide to download all results by checking the first selection box. Then a download button will appear at the bottom of the page in the right corner. Clicking on this button will bring up the download view. The download button will generate and download gene expression profiles and signatures selected within the search results as .csv files, and will also include the metadata information associated with the profiles in a zipped folder. an example is shown in Figure 4.

### 3.6. Files

**Figure 5:**
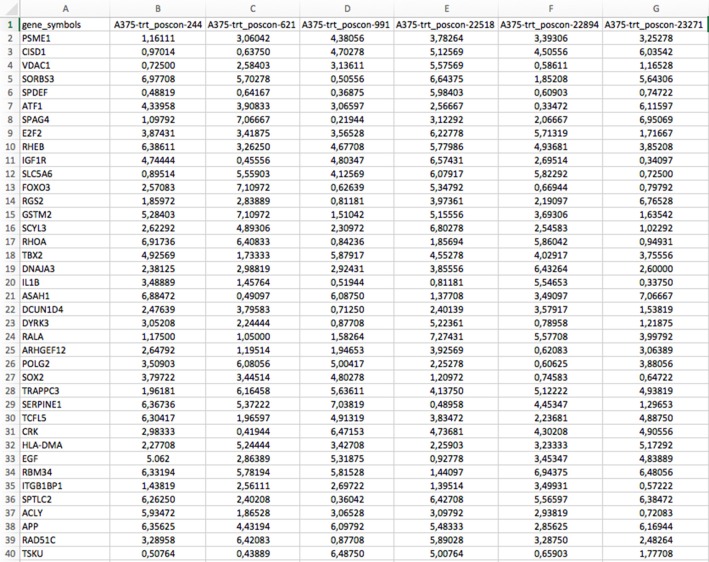
Example for a gene expression profile matrix (matrix.csv) available in the download zip folder.

**Figure 6:**
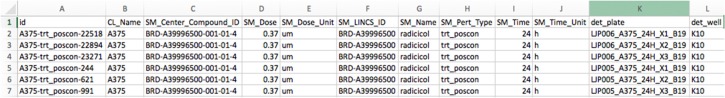
Example for a metadata matrix (metadata.csv) available in the download zip folder.

There are two files generated that are available in the zipped folder. The first is a comma-separated data matrix file (.csv) named “matrix.csv.” It contains the gene expression profiles of the downloaded dataset as shown in Figure 5. The rows in the file correspond to all the gene symbol annotations for each profile and the columns correspond to the samples. A second file contains the meta description of the profiles. It is also a comma-separated file named “metadata.csv.” This file contains the meta-information of the experiment of each profile, such as time points, dosages, profile IDs, etc. The content of the file is shown in Figure 6.

### 3.7. Data Visualization: An Example

In addition to the above search and downloading capability of our L1000 viewer, described above, we provide a graphical summarization of the selected files. Specifically, we provide code that can be used to plot (in an R environment) the statistical distributions of cell lines, dosage concentrations or time points. A user can make use of the scripts to visualize the data obtained directly from a specified query.

**Figure 7:**
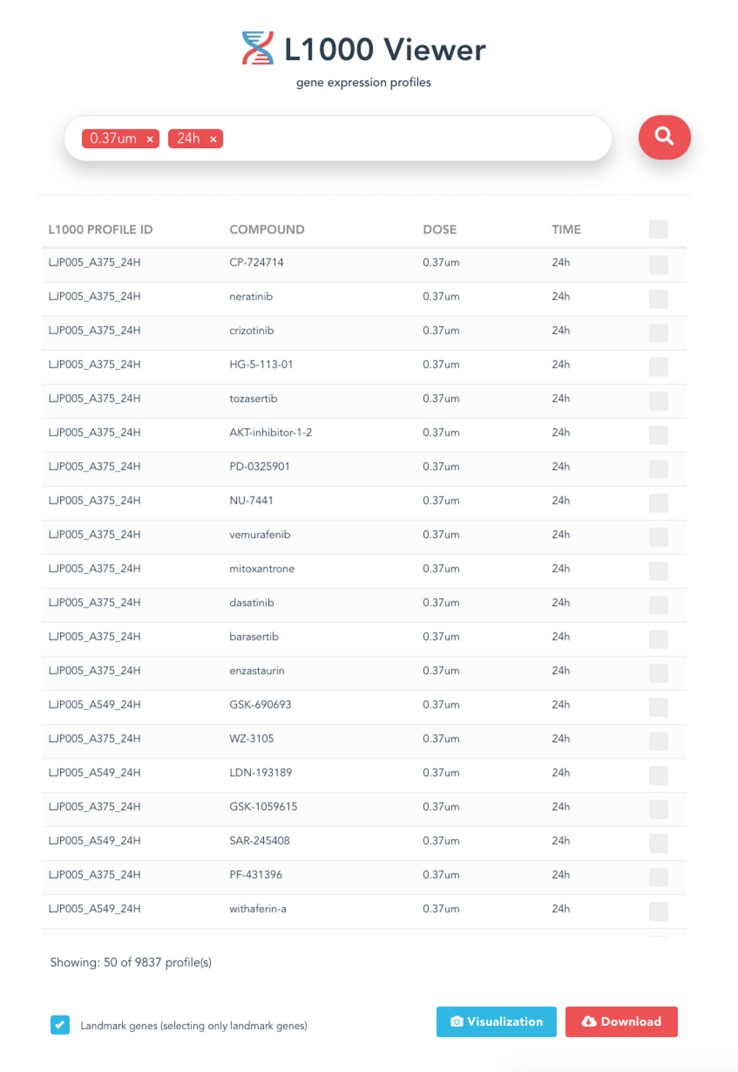
Sample query displaying drug profiles that are treated on different cell lines with 0.37 um concentration and 24 h time point. In total, 9,837 profiles have been retrieved.

**Figure 8:**
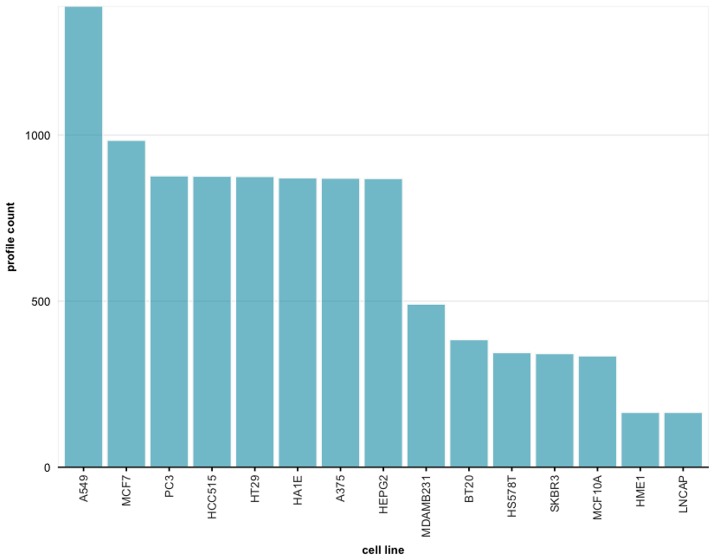
Frequency distribution for cell lines across all experiments retrieved from the query in Figure 7.

For instance, from the query shown in Figure 7, setting the concentration to 0.37um and the time points to 24h, 9, 837 profiles are obtained. In Figure 8 we show the distribution of these 9, 837 profiles over 15 cell lines. Here we leverage the metadata annotations downloaded along with the expression profiles obtained from the Cell Service API to show the distribution of each cell line.

**Figure 9:**
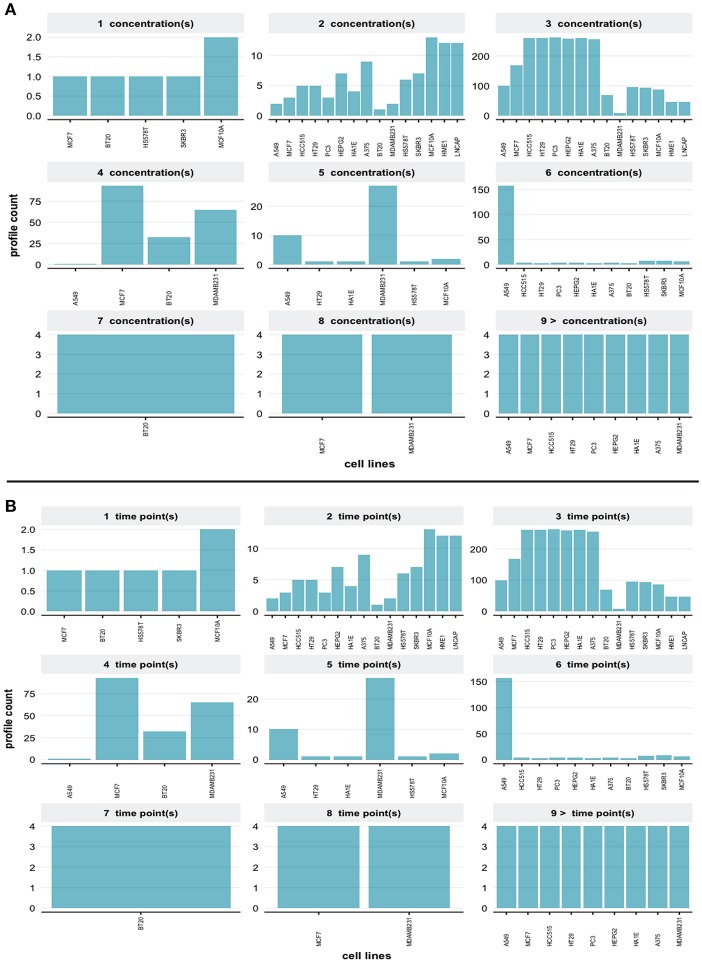
**(A)** Distributions of different dosages (concentrations) of small molecules for the query in Figure 7. **(B)** Distributions of different time points for the query in Figure 7.

eFor the same query we obtain the distribution of different concentrations of small molecule perturbagens, shown in Figure 9A. One can see that there are more than 9 different concentrations available in this data set. The compound information for small molecule perturbagens was retrieved using the Pert service API to identify unique and common compounds used in the L1000 data.

Finally, in Figure 9B we show the distribution of available time points in the data set. The R code and guidelines are provided from the web interface in order to subset and visualize the L1000 dataset using user specific query.

### 3.8. L1000 Viewer Accessibility

Access to the data indexed by the L1000 Viewer is provided through our web interface via http://L1000viewer.bio-complexity.com/. It enhances the biomedical data repository by providing a simple and fast access to LINCS raw data and allows to easily generate subsets of data. In this way, users of the web interface can extract knowledge more efficiently when interfacing with LINCS data.

### 3.9. Code Availability

All code associated with the L1000 Viewer project is open source. The code is available from the BitBuket repository (https://bitbucket.org/aliocee/devcrew/src/master/). The L1000 Viewer libraries are versioned according to the Semantic Versioning 2.0.0 guidelines (http://semver.org/).

## 4. Discussion

Advances in experimental and computational methods in biomedical research are now producing large volumes of digital data objects that are rapidly accumulating. At the same time, a variety of bioinformatics tools to handle the analysis of all this data are promptly being developed and published. However, systematic linking of digital data entities for easy access are currently lacking most especially for the LINCS L1000 raw data. That means there is a gap between the data availability and how much of it can be employed in applications for extracting useful knowledge.

Previous attempts to build gene expression content-based databases have provided new support for perturbational data accessibility (Subramanian et al., 2017; Wang et al., 2018). The data within these databases is structured, and thus suitable for data access; however, most attempts to represent such data only succeeded in accomplishing this in a complex representation. For example, web-based platforms such as the CLUE Platform (Li et al., 2019), LINCS Data Portal (Koleti et al., 2017), L1000FWD (Wang et al., 2018) or iLINCS (Keenan et al., 2018) provide information about signature profiles and metadata, but there are no easy-to-use resources that enable the user to access selected raw data. Specifically, the CLUE Platform is one of the most comprehensive resources for collective knowledge about the LINCS project and L1000 data, aggregating information from over 20, 000 perturbagens and 400, 000 signature profiles. However, the CLUE Platform is very complex and does not provide direct access to the raw data. Instead, it provides an open and free API for accessing metadata. Moreover, most of these platforms operate on metadata like annotated cell lines, proteins, and small molecules. Still they lack the simplicity and interactivity for users to access the data (Vempati et al., 2014). In comparison, our L1000 Viewer provides an easy-to-use interface for searching and downloading raw data.

The L1000 Viewer web application will enable the user to easily search the LINCS L1000 raw data via an interactive web interface. The L1000 Viewer is built using Javascript libraries, and is deployed as a Node.js application (Tilkov and Vinoski, 2010) in order to provide quick access. Its front end interface utilizes the core Vue.js libraries (You, 2017) and all gene expression and metadata are stored in a MongoDB database. Furthermore, we developed and integrated an API in our application that enables users to search the LINCS data repository and to automatically generate data for download.

In contrast to stand-alone software that needs to be installed locally on a computer, our L1000 Viewer is a web application that can be accessed via any web browser without the need of installing software on a computer locally. This makes it not only easy to access but ensures also an operating system independent functioning.

## 5. Conclusion

In this paper, we introduced the L1000 Viewer (http://L1000viewer.bio-complexity.com/), a search engine and graphical web interface for the LINCS data repository. The core of our L1000 Viewer is a database that utilizes the intricate dependency structure among the files in the LINCS data. This resulted in a reorganization of the files and enables efficient search capabilities based on graph-oriented operations.

Overall, the L1000 Viewer provides a useful tool for efficiently accessing exclusive information from the LINCS data repository that can be utilized for computational pharmacogenomics studies (Hopkins, 2008; Davis and Chawla, 2011; Emmert-Streib et al., 2013; Himmelstein et al., 2017), e.g., for drug repurposing and cancer therapeutics, as well as for understanding the composition and relationships between genes, drugs and diseases.

## Data Availability

Publicly available datasets were analyzed in this study. This data can be found here: https://www.ncbi.nlm.nih.gov/geo/query/acc.cgi?acc=GSE70138.

## Author Contributions

FE-S conceived this study. AM and ST performed the analysis. AM, ST, MD, and FE-S wrote the paper and approved the final version of the manuscript.

### Conflict of Interest Statement

The authors declare that the research was conducted in the absence of any commercial or financial relationships that could be construed as a potential conflict of interest.
